# Advancements in the Research of New Modulators of Nitric Oxide Synthases Activity

**DOI:** 10.3390/ijms25158486

**Published:** 2024-08-03

**Authors:** Cristina Maccallini, Roberta Budriesi, Barbara De Filippis, Rosa Amoroso

**Affiliations:** 1 Department of Pharmacy, University “G. d’Annunzio” of Chieti-Pescara, Via dei Vestini 31, 66100 Chieti, Italy; barbara.defilippis@unich.it (B.D.F.); rosa.amoroso@unich.it (R.A.); 2 Department of Pharmacy and Biotechnology, Food Chemistry and Nutraceutical Lab, Alma Mater Studiorum-University of Bologna, 40126 Bologna, Italy; roberta.budriesi@unibo.it

**Keywords:** activators, cancer, cardiovascular diseases, drug design, inflammation, inhibitors, natural sources, nitric oxide, nitric oxide synthase, neurodegenerative diseases

## Abstract

Nitric oxide (NO) has been defined as the “miracle molecule” due to its essential pleiotropic role in living systems. Besides its implications in physiologic functions, it is also involved in the development of several disease states, and understanding this ambivalence is crucial for medicinal chemists to develop therapeutic strategies that regulate NO production without compromising its beneficial functions in cell physiology. Although nitric oxide synthase (NOS), i.e., the enzyme deputed to the NO biosynthesis, is a well-recognized druggable target to regulate NO bioavailability, some issues have emerged during the past decades, limiting the progress of NOS modulators in clinical trials. In the present review, we discuss the most promising advancements in the research of small molecules that are able to regulate NOS activity with improved pharmacodynamic and pharmacokinetic profiles, providing an updated framework of this research field that could be useful for the design and development of new NOS modulators.

## 1. Introduction

Just over 25 years ago, the Nobel Prize won by Robert F. Furchgott, Louis J. Ignarro and Ferid Murad for their independent discoveries related to the role of nitric oxide (NO) in the cardiovascular system, definitively established the importance of this molecule in living systems, so much so that it was defined the “miracle molecule” [1]. In the beginning, the chemical structure of this powerful biological messenger was unknown, so it was reported as an endothelial relaxing factor. Later, it was identified as NO, deeply impacting different research fields [2], with about 53,000 articles with NO in the title published in the 2000′s that have improved our understanding of its mechanism of action and biosynthesis.

**Scheme 1:**
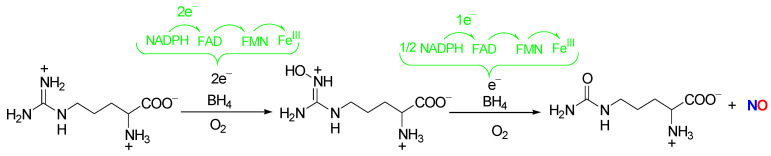
Global catalytic mechanism of NO synthesis. In the first step, L-Arg hydroxylation occurs in the presence of one molecule of oxygen and one equivalent NAPH via a sequential hydride and electron transfer through FAD, FMN up to heme and BH4. The formed N-hydroxyl-arginine requires a molecule of oxygen and one electron from 0.5 NADPH to be further oxidized to L-Citr and NO, and the final production of two H_2_O molecules. Abbreviations: NADPH: nicotinamide adenine dinucleotide phosphate; FAD: flavin adenine dinucleotide; FMN: flavin mononucleotide; and BH4: tetrahydrobiopterin.

Nitric oxide synthases (NOSs) are a family of enzymes responsible for the production of NO. They use L-arginine (L-Arg) as a substrate, converting it into NO and L-citrulline (L-Citr) through a series of oxidation reactions (Scheme 1). This process requires several cofactors, including nicotinamide adenine dinucleotide phosphate (NADPH), flavin adenine dinucleotide (FAD), flavin mononucleotide (FMN), and tetrahydrobiopterin (BH4) [3] The activity of NOS enzymes is tightly regulated at multiple levels, including gene expression, protein–protein interactions, post-translational modifications, as well as by the availability of its substrate and cofactors.

**Figure 1:**
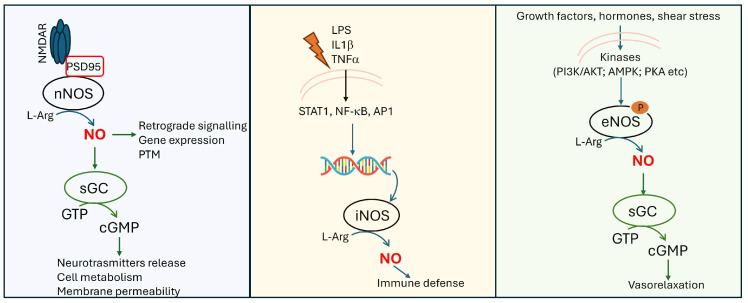
NOS isoforms and their regulation and effects. The nNOS is activated by its interaction with the NMDAR/PSD95 complex. The generated NO interacts with the sGC, which catalyzes the production of cGMP from the GTP, and then the cGMP mediates downstream effects. The transcription of iNOS is mediated by different proteins, such as NF-κB, STAT1, and AP1, in response to proinflammatory stimuli. NO is generated at high levels as an immune defense. In physiological conditions, eNOS is activated through its phosphorylation by different protein kinases, and the produced NO, by interacting with the sGC, maintains vascular homeostasis. Abbreviations: NMDAR: N-methyl-D-aspartate receptor; PSD95: postsynaptic density protein 95; sGC: soluble guanylate cyclase; cGMP: cyclic guanosine monophosphate; GTP: triphosphate guanosine; PTM: post-translational modifications; LPS: lipopolysaccharides; IL1β: interleukin-1β; TNFα: tumor necrosis factor α; NF-κB: nuclear factor kappa-light-chain-enhancer of activated B cells; STAT1: signal transducer and activator of transcription 1; AP1: activator protein 1; PI3K/AKT: phosphatidylinositol 3-kinase/protein kinase B; AMPK: 5′AMP-activated protein kinase; and PKA: protein kinase A.

There are three main isoforms of NOSs, two of which are constitutive, i.e., the neuronal (nNOS or NOS1) and the endothelial (eNOS or NOS3), and one that is inducible (iNOS or NOS2). Each of these isoforms plays distinct roles in the body and is regulated differently (Figure 1). The nNOS is primarily found in the nervous system, and it is anchored to the neuronal postsynaptic membrane by means of the postsynaptic density protein 95 (PSD-95). When N-methyl-D-aspartate receptors (NMDARs) are stimulated, a ternary NMDAR/PSD95/nNOS complex is formed, initiating nNOS activity [4]. The NO produced plays different roles, involved in neurotransmission and the regulation of synaptic plasticity, which is essential for learning and memory and affects neurogenesis [5]. Additionally, it contributes to the regulation of blood flow and muscle contraction. The eNOS is predominantly found in endothelial cells, which line the blood vessels, and can be activated by different signals such as growth factors, hormones, and shear stress that stimulate the eNOS phosphorylation [6]. NO produced by eNOS plays a critical role in maintaining vascular homeostasis by regulating vascular tone, blood pressure, and blood flow. Moreover, it inhibits platelet aggregation and prevents the adhesion of leukocytes to the endothelium, thus protecting against atherosclerosis and thrombosis [7].

The iNOS is induced in response to inflammatory signals, such as cytokines and bacterial endotoxins. It produces large amounts of NO as part of the immune response, helping to kill pathogens and modulate inflammation. While this can be beneficial in fighting infections, excessive iNOS activity can lead to tissue damage and contribute to inflammatory diseases [8]. 

Several studies have been reported pointing to the dual roles of NO in both normal physiology and disease states, and understanding this ambivalence is crucial for medicinal chemists to develop therapeutic strategies that regulate NO production without compromising its beneficial functions in cell physiology. The different effects of NOSs are related to different factors, and one major aspect is the level of NO produced. This depends not only on the specific activated NOS isoform but also on the bioavailability of the cofactors, as well as the extension of the NOS expression and its appropriate activation [6,7,8]. In this review, we focus on the advancements and critical issues in the development of therapeutic options related to the modulation of NOS activity by means of small molecules.

## 2. The NOS Structure

When targeting NOS activity, a well-defined issue is isoform selectivity. There is no doubt that a given inhibitor must act without modifying the activity of eNOS and that, in general, modulating the activity of one or more isoforms depends on specific pathological conditions. Therefore, studies aimed at highlighting the structural differences between NOSs have been of fundamental importance for the design of molecules that are able to selectively bind to a single enzyme. 

**Figure 2:**
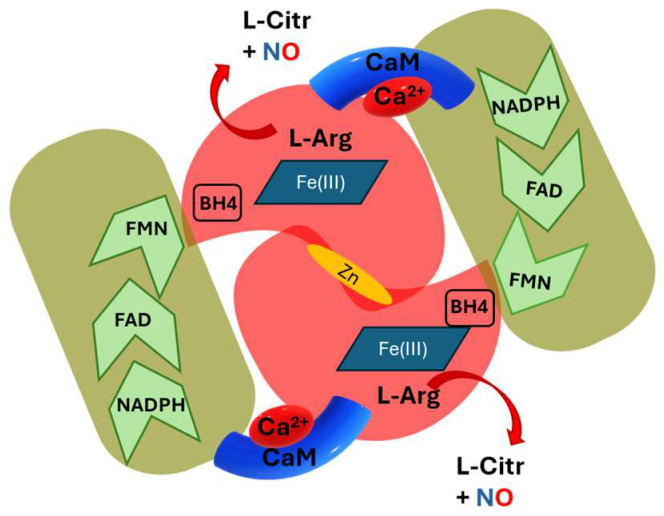
Schematic representation of the NOS dimer. Each monomer is formed by the oxygenase domain (in red) containing the heme and the BH4 and L-Arg-binding sites and the reductase domain (in green) containing the cofactor binding sites. The oxygenase domain is bound to the reductase domain by a calcium–calmodulin (Ca^2+^-CaM) binding sequence. Zn–tetrathiolate is bound at the dimer interface between the two oxygenase domains.

NOSs have a complex structure consisting of a homodimer in which each subunit is approximately 130–150 kDa in size [9]. The enzyme operates through a two-step process involving the following two primary domains: the oxygenase domain and the reductase domain, which are connected by a calmodulin-binding region (Figure 2). The oxygenase domain is located at the N-terminal of the NOS protein. This domain is responsible for binding the substrate L-arginine and converting it to nitric oxide and L-citrulline. Key structural features of the oxygenase domain include the following: (i) a heme prosthetic group, which is essential for its catalytic activity. The heme iron is the site where oxygen is activated, and NO synthesis begins, and it participates in L-Arg oxidation through complex mechanisms that are not fully elucidated [2,3,10]. (ii) BH4 is a critical cofactor that stabilizes the enzyme and aids in the electron transfer necessary for NO production [11]; finally, (iii) the substrate binding site is the site that specifically binds L-Arg, positioning it correctly for the catalytic reaction to occur [3]. Many NOS inhibitors developed so far compete with L-Arg, blocking enzymatic activity [12].

The calmodulin-binding region is located between the oxygenase and reductase domains. This region is crucial for the regulation of NOS activity. Calmodulin binding increases the efficiency of electron transfer by constraining the rotational movements of the reductase domain relative to the oxidase domain. In particular, CaM binding releases the FMN subdomain from the FAD/NADPH subdomain, allowing electron transfer to the oxidase domain [13]. Unlike neuronal and endothelial NOSs, which are regulated by calcium/calmodulin binding, iNOS is constitutively bound to calmodulin once it is expressed. This binding ensures that iNOS is fully active under conditions of inflammation or immune response [14,15].

The reductase domain is located at the C-terminal of the NOS protein. This domain is responsible for transferring electrons from NADPH to the oxygenase domain to facilitate NO production. Besides NADPH, the reductase domain includes several important cofactors as follows: FAD, which accepts electrons from NADPH and passes them to FMN, which then transfers them to the heme group in the oxygenase domain. It was observed that one reductase domain at a time contributes to NOS catalysis, with the other remaining in the input state [13].

The hinge region connects the oxygenase and reductase domains and provides flexibility for the enzyme to undergo conformational changes necessary for electron transfer between the domains. This region is also involved in the calmodulin-mediated regulation of iNOS activity [16]. 

The dimerization interface is crucial for the formation of the active dimeric structure of NOS. This interface is formed by interactions between the oxygenase domains of the two subunits, stabilizing the enzyme and optimizing its catalytic efficiency [17].

NOS isoforms exhibit a high degree of sequence homology, reflecting their evolutionary relationship and functional similarities. However, the exact grade of homology varies depending on the specific regions of the enzymes being compared. Variations in the aminoacidic sequence are most prominent in regions critical for substrate and cofactor binding, enzyme dimerization, and interactions with regulatory proteins. For example, the oxygenase domains of the three isoforms contain conserved regions for the substrate catalytic pocket, the heme, and BH4 binding; yet, amino acids in the substrate access channel vary, affecting the binding affinities and catalytic efficiencies for each isoform [18,19]. Therefore, NOS inhibitor-bearing moieties that were able to interact with these regions were developed in order to obtain select compounds.

Moreover, the specific calcium/calmodulin-binding motifs that are included in the nNOS and eNOS structures allow their activities to be tightly regulated by intracellular calcium levels, whereas iNOS lacks these motifs, leading to its calcium-independent, constitutive activation once expressed [20]. The reductase domains also exhibit subtle sequence variations that influence their electron transfer rates and interactions with other cofactors. Additionally, some differences among the isoforms can be found at the dimerization interfaces, with eNOS having a more stable interface [21]. These amino acid sequence variations enable each NOS isoform to adapt its structure and function to meet specific physiological demands, and, in principle, they can be exploited to design selective modulators. However, it should be considered that there are some differences in the expression and regulation of NOSs in human versus animal cell models, both in physiologic and disease conditions [22,23]. Considering this issue, appropriate preclinical evaluations should be performed. 

## 3. nNOS Modulators 

The dysregulation of nNOS is implicated in various neurological conditions, including stroke, neurodegenerative diseases, and neuropathic pain. In particular, in the early stages of pathological conditions, there is often an overactivation of the NMDARs [24]. This results in an excessive influx of calcium ions (Ca^2+^) in the consequent nNOS-extended overstimulation. The excess NO produced during this process contributes to disease progression by mediating the post-translational modifications (PTMs) of proteins, such as nitrotyrosination (Tyr-NO2), which involves the reaction of tyrosine residues with peroxynitrite (ONOO−), and cysteine nitrosylation (SNO) [25]. In these disease contexts, PTMs are primarily pathological, leading to either the loss or gain of protein function. Therefore, nNOS inhibition is regarded as a potential therapeutic option, and nNOS inhibitors could be useful to rebalance NO production. Small molecules that are able to selectively inhibit the nNOS can act by binding specific enzymatic sequences located at the L-Arg binding site or preventing the interaction of nNOS with the postsynaptic density protein 95 (PSD-95), which anchors nNOS to the postsynaptic membrane in neurons and is crucial for the enzyme‘s activation. These molecules have been mainly studied at the preclinical and clinical levels, as reported in Table 1.

**Figure 3:**
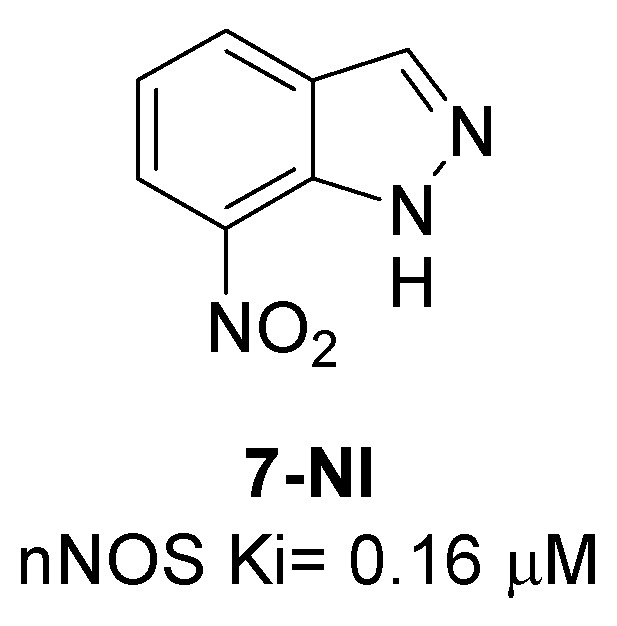
Chemical structure of 7-nitroindazole.

There are several examples of selective small molecules targeting the nNOS binding site. One of the most used pharmacological tools to study the therapeutic effects of nNOS inhibition is the 7-nitroindazole (7-NI, Figure 3), which provides neuroprotection in different disease models [26,27,28].

**Figure 4:**
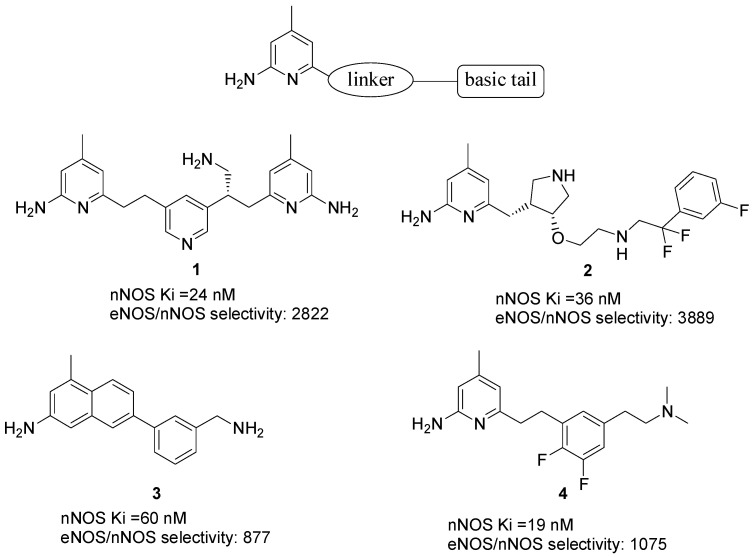
General pharmacophoric model of 2-aminopyridine-based nNOS inhibitors and the chemical structure of selected derivatives **1**–**4**.

This molecule is a quite effective nNOS inhibitor (Ki = 0.16 μM), although it does not exhibit isoform selectivity in vitro [29]. This could be due to its mechanism of action, which involves binding to the NOS’s heme by the iron chelation [29]. However, some in vivo studies reported that 7-NI does not affect blood pressure since it does not inhibit eNOS; therefore, it was suggested that this molecule could be accumulated in the nervous system, resulting in tissue selectivity [30]. Nevertheless, a full consensus regarding 7-NI’s in vivo selectivity has never been reached [30]. A promising class of molecules able to selectively inhibit nNOS activity by targeting the enzyme’s catalytic site is based on the 2-aminopyridine scaffold. In these compounds, different lipophilic and basic moieties were connected to the pyridine core by means of a central linker, which is often a cycle (Figure 4), with the aim to modulate their potency of action and selectivity towards nNOS, as well as their pharmacokinetic properties and blood–brain barrier permeation [31,32,33,34].

The most promising molecule is **4**, which showed excellent potency for rat (Ki = 15 nM) and human nNOS (Ki = 19 nM), with a selectivity of 1075-fold over human eNOS and 115-fold over human iNOS. Moreover, **4** displayed excellent cell-membrane permeability and good metabolic stability. A crystallographic study clarified the binding mode of these molecules in the human nNOS. The 2-aminopyridine binds with a bidentate, H-bonding the active site Glu residue. The presence of the 4-methyl group ensures further non-polar interactions with a small pocket close to the active site. The central aromatic ring is positioned over the heme, assuming a specific upward orientation, which places the amine tail towards a H_2_O molecule positioned in between the BH4 and the heme propionate A. This typical binding mode is different in the eNOS active site. Here, different interactions are lost since the central aromatic core is less favorably orientated over the heme so that also the amine tail cannot establish interactions. Very interestingly, it was found that bis-2-aminopyridine derivatives, such as compound **1**, as well as 2-aminoquinoline-based compounds, such as **3**, are able to bind to both the active site, as expected, and the BH4 pocket. Crystallographic studies revealed that two molecules bind to each dimer subunit and that the second can displace the BH4 cofactor in nNOS, although this is much less likely to occur in eNOS [35]. This is the reason for the high selectivity over the eNOS exhibited by these molecules.

**Table 1:** Inhibitors of the nNOS evaluated in preclinical/clinical studies.

Compound	Target	Therapy Area	Study	Reference
7-NI	NOS heme	neuroprotection	preclinical	[26,27,28]
**1**–**4**	L-Arg catalytic site	neurodegeneration/ neuropathic pain	preclinical	[34]
AVLX-144	nNOS-PSD95 interaction	stroke	Phase 1 (completed)	[36]
ZL006	nNOS-PSD95 interaction	stroke	preclinical	[37,38,39]
SCR4026	nNOS-PSD95 interaction	stroke	preclinical	[40]
**5**	nNOS-PSD95 interaction	stroke	preclinical	[41]
nerinetide	NMDAR-PSD95 interaction	stroke	Phase III (completed)	[42]

**Figure 5:**
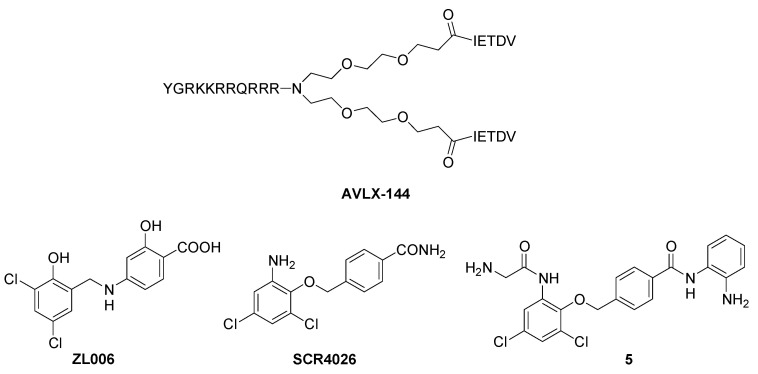
Chemical structure of nNOS-PSD95 interaction inhibitors.

On the other side, few examples are reported of compounds able to block the nNOS-PSD95 interaction, which is vital for the precise spatial production of NO. This interaction occurs among the nNOS PDZ (postsynaptic density-95, discs large, zonula occludens-1) domain, located at the N-terminus of nNOS, and the PSD-95′s PDZ2 subunit and can be blocked either by small molecules or peptides. In general, these compounds do not affect the NMDA receptor function, nNOS expression, or nNOS catalytic activity, and for this reason, can be considered safer with respect to molecules directed against the nNOS active site. AVLX-144 (Figure 5) is a peptide-based molecule under clinical investigation with promising activity in neurodegeneration and stroke models [36]. It binds the PSD95′s PDZ1 and PDZ2 domains simultaneously, preventing interactions with both NMDARs and nNOS. Small molecules based on a benzyl-aniline scaffold are able to bind to the nNOS PDZ domain, blocking protein–protein interactions. For example, ZL006 (Figure 5) diminishes the neuronal damage and oxidative stress caused by Aβ1-42 and reduces neuronal injury and apoptotic cell death in a model of Parkinson’s disease [37,38]. Moreover, it has demonstrated promising neuroprotectant activity in stroke in vivo models [39]. SCR4026 (Figure 5) exhibited neuroprotective activities and showed potential in treating ischemia stroke by disrupting PSD95-nNOS protein–protein interactions. However, it displayed moderate solubility and short half-time [40], and these issues were optimized by the introduction of a 2-aminobenzamide and a glycine group on the aniline of the SCR4026, obtaining compound **5** (Figure 5) [41]. The last showed significant neuroprotective effects on damaged primary cultured neurons at 0.1 μM. Moreover, it had therapeutic effects in the MCAO rat model, reducing the era of ischemic cerebral infarction and improving rats’ behavior.

Very recently, a phase III clinical trial (ESCAPE-NA1) was completed on the drug candidate nerinetide in the treatment of acute ischemic stroke [42]. This is an eicosapeptide that prevents the NMDAR/PSD-95 interaction and, consequently, inhibits the neurotoxic signaling of nNOS. The results from this study showed no significant differences between placebo- and nerinetidine-treated patients at 90 days after stroke, and this was related to the cleavage of nerinetide by plasmin, which is activated by the co-administration of the tissue plasminogen activator alteplase. Therefore, this trial encourages the development of other neuroprotective drugs targeting the NMDAR/PSD95/nNOS activation. 

## 4. iNOS Modulators

Dysregulated iNOS contributes to the pathology of chronic inflammatory diseases, such as rheumatoid arthritis [43], inflammatory bowel disease [44], and asthma [45]. In neurodegenerative disorders like Parkinson’s and Alzheimer’s disease, excessive NO can exacerbate neuronal injury and death [46]. Additionally, iNOS-derived NO is involved in the pathogenesis of certain cancers by influencing tumor progression, angiogenesis, and immune evasion [47,48,49]. Table 2 provides a list of those promising selective iNOS inhibitors discussed in this section with proven therapeutic action. 

**Figure 6:**
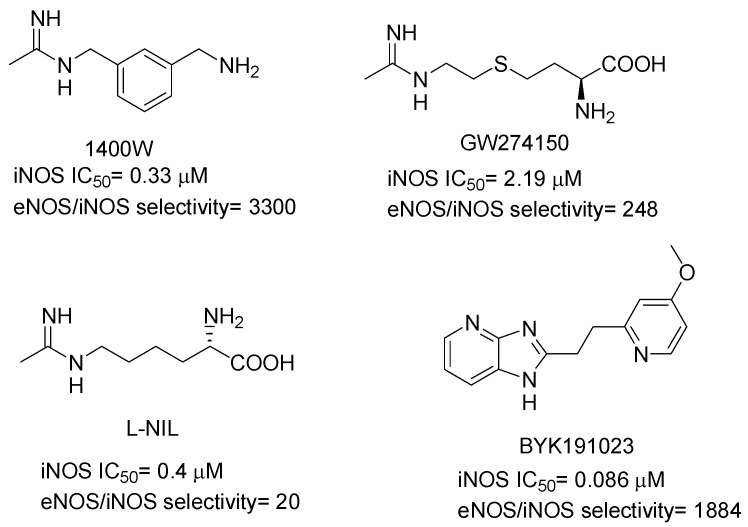
Chemical structures of the first generation of potent and selective iNOS inhibitors.

Several compounds able to target the iNOS have been reported during the 2000′s, and some of them resulted in appearing very potent and selective, such as 1400W, GW274150, L-NIL (N6-(1-iminoethyl)-L-lysine hydrochloride), and BYK191023 (Figure 6) [50,51,52,53] In particular, these compounds are competitive inhibitors, binding to the iNOS catalytic site, and they proved to be a useful pharmacological tool to investigate the effects of iNOS inhibition in different pathological contexts, such as in stroke [54], hyperalgesia [55] cancer and cancer chemo-resistance [56], septic shock [57], renal ischemia/reperfusion injury [58], Parkinson’s disease [59], and rheumatoid arthritis [60]. 

Despite these promising results, none of these compounds progressed into advanced clinical trials, mainly due to preclinical toxicity or failure to slow the disease progression [61,62,63]. One of the reasons for these unsuccessful trials might be differences in the disease patho-mechanism among different species so that preclinical animal models are not sufficiently predictive [23]. Other issues are inappropriate for the in vivo iNOS inhibition degree, as well as the lack of safety and tissue specificity exhibited by the known molecules [64]. Therefore, the research of new iNOS modulators is highly desired, and different interesting molecules have been recently reported. 

**Figure 7:**
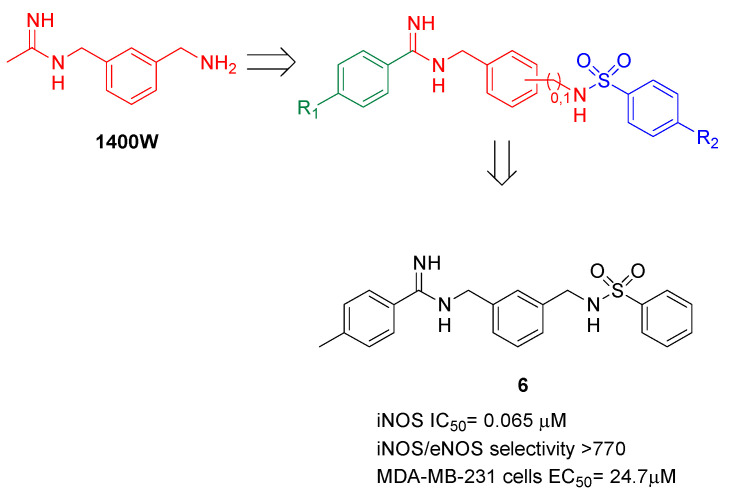
The general structure of phenyl-amidine-based compounds related to 1400W and the chemical structure of the most interesting molecule, **6**. In the general structure, the phenyl ring, which was introduced to ameliorate 1400W lipophilicity, is reported in green; in red, the 1400W amidine-benzyl core is reported, and in blue, the phenyl-sulfonamide moiety responsible for the compounds’ selectivity is shown.

Based on the leading structure of the 1400W, a set of phenyl-amidine derivatives bearing a sulfonamide moiety was prepared (Figure 7), and molecule **6** gave promising results in terms of its potency of action (human iNOS IC_50_ = 0.65 μM), selectivity over constitutive isoforms, and antiproliferative and antimetastatic effects in a triple-negative breast cancer cell line [65]. A docking study revealed that the sulfonamide group interacted with the iNOS-specific Asp382, stabilizing benzamidine binding on the catalytic site. This interaction is lost in the eNOS, where the molecule is forced toward the opposite side of the catalytic site entrance, disrupting the effectiveness of benzamidine interactions.

**Table 2:** Inhibitors of the iNOS evaluated in preclinical/clinical studies.

Compound	Target	Therapy Area	Study	References
1400W	L-Arg catalytic site	Hyperalgesia, stroke, cancer	preclinical	[54,55,56]
GW274150	L-Arg catalytic site	Septic shock, neuroprotection, rheumatoid arthritis	Phase 2 (completed)	[59,60]
L-NIL	L-Arg catalytic site	renal ischemia/ reperfusion injury	preclinical	[58]
BYK191023	L-Arg catalytic site	septic shock; cancer	preclinical	[57]
**6**	L-Arg catalytic site	cancer	preclinical	[65]
**7**–**8**	L-Arg catalytic site	inflammation	preclinical	[66,67]
**9**–**12**	Inhibition of NF-kB	inflammation, cancer	preclinical	[68,69,70,71]
**13**–**15**	iNOS dimerization	arthritis	preclinical	[72,73]
chrysamide B	iNOS dimerization	oedema, septic shock	preclinical	[74]

**Figure 8:**
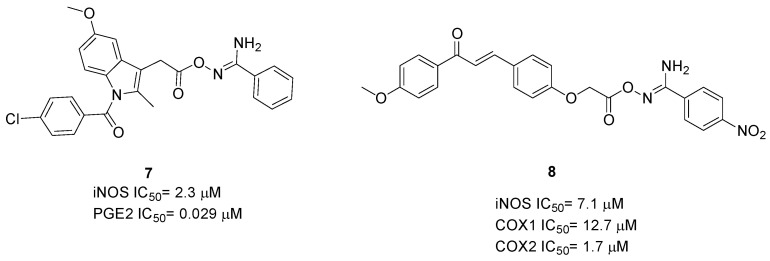
Aril-carboximidamide-based compounds for iNOS inhibitors endowed with anti-inflammatory activity.

Aril-carboximidamides **7**–**8** were reported for their capability to reduce iNOS-derived NO, as well as to inhibit PGE2 and COX in LPS-stimulated RAW264.7 macrophages (Figure 8). In these molecules, the imidamide group acted as an amidine bioisoster, and encouraging results were obtained in an in vivo model of carrageenan-induced rat paw odoema [66,67].

**Figure 9:**
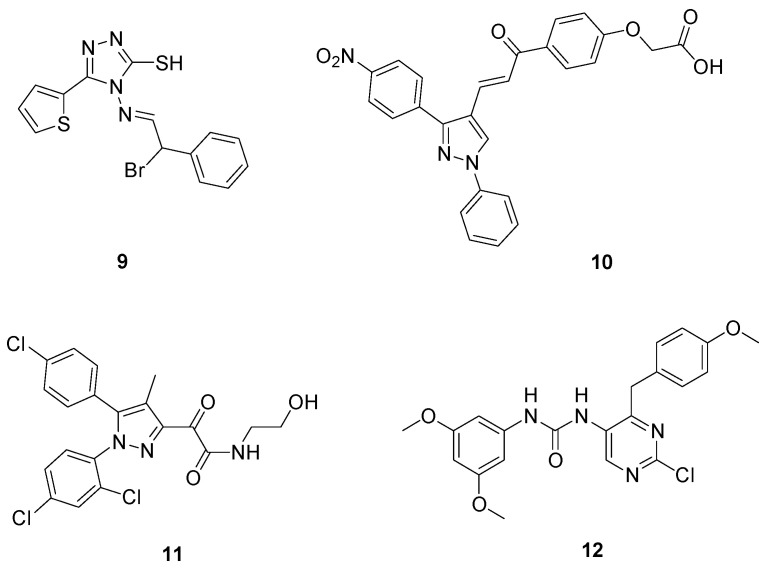
Chemical structures of iNOS inhibitors containing a nitrogen heterocycle.

Interestingly, compounds including a nitrogen heterocycle, such as triazole, pyrazole, or a pirimidine core, proved to be promising iNOS modulators. Compounds like **9**–**12** (Figure 9) mainly acted as anti-inflammatory agents able to modulate the NF-kB pathway in inflamed cellular models, such as in PAM212 keratinocytes and different cancer cells (**9**, **10**) [68,69], BV2 cells (**11**) [70], and RAW 264.7 macrophages (**12**) [71], resulting in a reduction in iNOS expression.

**Figure 10:**
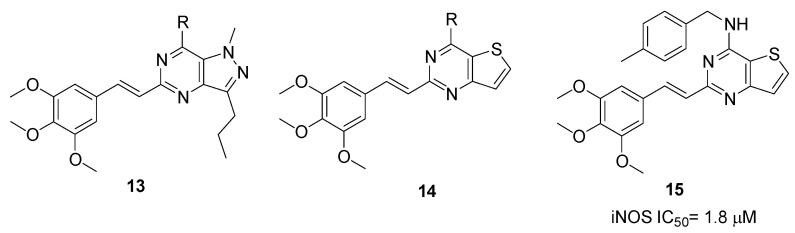
Chemical structure of iNOS dimerization inhibitors.

On the other side, molecules **13**–**15** (Figure 10) acted as iNOS dimerization inhibitors, i.e., small molecules preventing the assembly of the initially synthesized monomeric NOS protein into the functional homodimer [72,73]. In particular, the azole moiety binds to the heme, causing a conformational change in the monomer, which prevents the formation. These compounds do not suffer from the typical loss of cellular effectiveness of L-Arg competitive inhibitors, which is due to the high concentrations of the natural substrate. For example, thieno[3,2-d]pyrimidine derivative **14** inhibits the stabilization of iNOS dimers in LPS-stimulated RAW 264.7 cells, resulting in quite potent iNOS inhibition (IC_50_ = 1.86 μM). Its anti-inflammatory activity was assessed by an adjuvant-induced arthritis rat model in vivo, and it showed good drug-like properties. 

**Figure 11:**
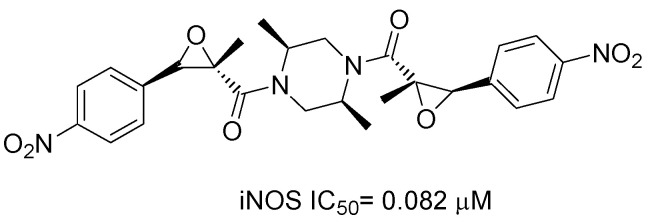
Chemical structure of chrysamide B, which is a potent natural iNOS dimerization inhibitor.

Chrysamide B (Figure 11) is a natural compound isolated from the deeper sea fungi Penicillium chrysogenum. It has a dimeric nitrophenyl trans-epoxyamide structure and has been demonstrated to inhibit the overproduction of NO and the activation of iNOS (IC_50_ = 0.082 μM) by affecting the formation of dimeric iNOS. Chrysamide B provided anti-inflammatory effects in carrageenan-induced paw edema and in LPS-induced septic mice and can be considered a promising chemical scaffold to obtain new iNOS inhibitors [74].

## 5. eNOS Modulators

The function and activity of eNOS in the endothelium are crucial for maintaining vascular integrity and homeostasis. Most cardiovascular diseases are associated with the imbalanced production of ROS and peroxynitrite, which, by oxidating the cofactor BH4, triggers the so-called eNOS uncoupling, which is a dysfunctional state where the enzyme produces superoxide instead of NO [75,76]. This shift contributes to even more oxidative stress and endothelial dysfunction, playing a significant role in the pathogenesis of various cardiovascular diseases [77]. Consequently, eNOS is an appealing therapeutic target that has yet to be pharmacologically utilized. eNOS enhancers that have demonstrated a therapeutic potential are reported in Table 3.

**Figure 12:**
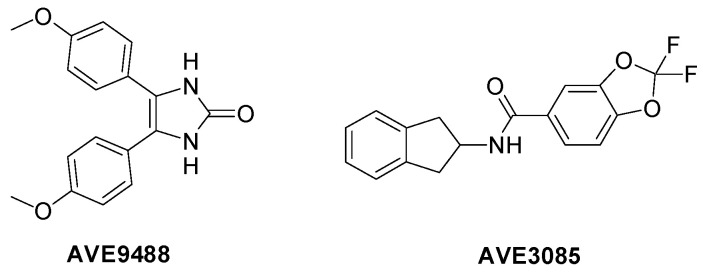
Chemical structure of the eNOS stimulators AVE9488 and AVE3085.

Compounds like AVE9488 and AVE3085 (Figure 12) are eNOS enhancers, upregulating the enzyme’s transcriptional levels. These molecules produced highly promising preclinical results, such as the improvement of left ventricular remodeling in myocardial infarction [78] and the improvement of diastolic heart failure in rats. In diabetic rats, AVE3085 normalized altered hind limb blood flow and reduced vascular inflammation. Additionally, this molecule restored endothelial function and blood pressure in spontaneously hypertensive rats and decreased oxidative stress and endothelial dysfunction in diabetic db/db mice [79,80]. Despite these results, human clinical trials were inconclusive, and these molecules were not entered into therapy. A possible reason for this failure is that the upregulated eNOS levels require increased BH4 levels at the same time; otherwise, eNOS uncoupling occurs. Therefore, the supplementation of BH4 or its precursors (e.g., sepiapterin or folate) could improve the clinical outcomes of eNOS activation [81,82].

**Figure 13:**
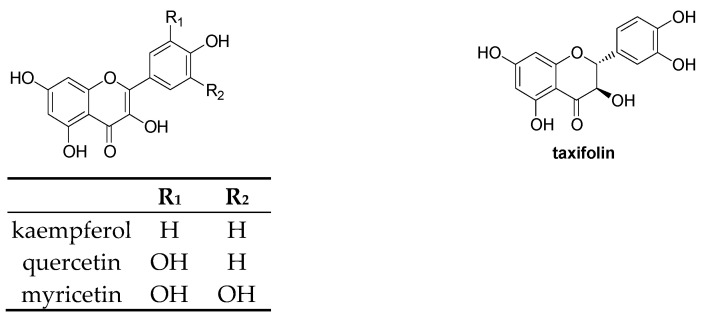
Chemical structure of flavonoids regulating the expression and the activity of eNOS.

Natural compounds, such as flavonoids (Figure 13), have undoubtedly attracted considerable interest for their capability to activate the eNOS, triggering the enzyme’s phosphorylation via the upstream activation of signals such as PI3K/Akt, or by regulating the p38, ERK1/2, MAPKs, and Nrf-2 pathways [83,84,85,86,87,88,89]. These compounds are also radical scavengers and inhibitors of ROS production, with the potential to improve BH4 availability in the inflammatory disease context [90,91]. Although dietary flavonoids were proven to preserve eNOS functions, limiting its uncoupling, this evidence was obtained in an animal preclinical model of cardiovascular diseases; therefore, more advanced clinical studies are necessary to ascertain their activity in human pathological conditions. 

**Table 3:** eNOS activators evaluated in preclinical studies.

Compound	Target	Therapy Area	Study	Reference
AVE9488; AVE3085	transcription enhancers	cardiac remodeling; endothelial dysfunction, hypertension	preclinical	[78,79,80]
taxifolin	eNOS phosphorilation	hypertension	preclinical	[89]
kaempferol	eNOS phosphorilation	wound healing	preclinical	[85]
quercetin	eNOS phosphorilation	endothelial dysfunction	preclinical	[86,87]
myricetin	eNOS phosphorilation	endothelial dysfunction	preclinical	[88]

Another issue is polymorphisms in gene-encoding eNOS, which can affect the enzyme’s regulation, thereby impacting responses to eNOS enhancers [92]. In this regard, pharmacogenetic studies are highly requested to appropriately define the potential usefulness of eNOS enhancers.

## 6. Conclusions

Since their identification, NOSs have gained considerable interest as a pharmacological target to treat different conditions, from cancer to neurodegenerative diseases, from septic shock to metabolic syndrome, and from migraine to autoimmune and cardiovascular diseases. Crystallographic and biochemical studies have revealed the NOS architecture, and the initial discovery of many NOS ligands has largely contributed to understanding the subtle differences among the three NOS isoforms. These studies have led to the improved design of selective NOS modulators, and the identification of these issues has also limited the progression of NOS modulators into clinics due to inappropriate safety, irreproducible outcomes from animal models, and isoform polymorphism. In this review, a discussion of the most important findings about the modulators of the three NOS isoforms is provided, with a particular focus on those promising molecules recently reported that could be used to translate the promising preclinic evidence into therapeutic outcomes.

## References

[1] Smith O. (1998). Nobel Prize for NO research. Nat. Med..

[2] Moncada S., Higgs E.A. (2006). The discovery of nitric oxide and its role in vascular biology. Br. J. Pharmacol..

[3] Santolini J. (2011). The molecular mechanism of mammalian NO-synthases: A story of electrons and protons. J. Inorg. Biochem..

[4] Brenman J.E., Chao D.S., Gee S.H., McGee A.W., Craven S.E., Santillano D.R., Wu Z., Huang F., Xia H., Peters M.F. (1996). Interaction of nitric oxide synthase with the postsynaptic density protein PSD-95 and alpha1-syntrophin mediated by PDZ domains. Cell.

[5] Maccallini C., Amoroso R. (2023). Neuronal Nitric Oxide Synthase and Post-Translational Modifications in the Development of Central Nervous System Diseases: Implications and Regulation. Molecules.

[6] Qian J., Fulton D. (2013). Post-translational regulation of endothelial nitric oxide synthase in vascular endothelium. Front. Physiol..

[7] Govers R., Rabelink T.J. (2001). Cellular regulation of endothelial nitric oxide synthase. Am. J. Physiol. Renal Physiol..

[8] Cinelli M.A., Do H.T., Miley G.P., Silverman R.B. (2020). Inducible nitric oxide synthase: Regulation, structure, and inhibition. Med. Res. Rev..

[9] Förstermann U., Sessa W.C. (2012). Nitric oxide synthases: Regulation and function. Eur. Heart J..

[10] Zhu Y., Silverman R.B. (2008). Revisiting heme mechanisms. A perspective on the mechanisms of nitric oxide synthase (NOS), Heme oxygenase (HO), and cytochrome P450s (CYP450s). Biochemistry.

[11] Wei C.C., Crane B.R., Stuehr D.J. (2003). Tetrahydrobiopterin radical enzymology. Chem. Rev..

[12] Minhas R., Bansal Y., Bansal G. (2020). Inducible nitric oxide synthase inhibitors: A comprehensive update. Med. Res. Rev..

[13] Campbell M.G., Smith B.C., Potter C.S., Carragher B., Marletta M.A. (2014). Molecular architecture of mammalian nitric oxide synthases. Proc. Natl. Acad. Sci. USA..

[14] Venema R.C., Sayegh H.S., Kent J.D., Harrison D.G. (1996). Identification, characterization, and comparison of the calmodulin-binding domains of the endothelial and inducible nitric oxide synthases. J. Biol. Chem..

[15] Knudsen G.M., Nishida C.R., Mooney S.D., Ortiz de Montellano P.R. (2003). Nitric-oxide synthase (NOS) reductase domain models suggest a new control element in endothelial NOS that attenuates calmodulin-dependent activity. J. Biol. Chem..

[16] Jones R.J., Smith S.M., Gao Y.T., DeMay B.S., Mann K.J., Salerno K.M., Salerno J.C. (2004). The function of the small insertion in the hinge subdomain in the control of constitutive mammalian nitric-oxide synthases. J. Biol. Chem..

[17] Panda K., Rosenfeld R.J., Ghosh S., Meade A.L., Getzoff E.D., Stuehr D.J. (2002). Distinct dimer interaction and regulation in nitric-oxide synthase types I, II, and III. J. Biol. Chem..

[18] Ji H., Li H., Flinspach M., Poulos T.L., Silverman R.B. (2003). Computer modeling of selective regions in the active site of nitric oxide synthases: Implication for the design of isoform-selective inhibitors. J. Med. Chem..

[19] Garcin E.D., Arvai A.S., Rosenfeld R.J., Kroeger M.D., Crane B.R., Andersson G., Andrews G., Hamley P.J., Mallinder P.R., Nicholls D.J. (2008). Anchored plasticity opens doors for selective inhibitor design in nitric oxide synthase. Nat. Chem. Biol..

[20] Garcin E.D., Bruns C.M., Lloyd S.J., Hosfield D.J., Tiso M., Gachhui R., Stuehr D.J., Tainer J.A., Getzoff E.D. (2004). Structural basis for isozyme-specific regulation of electron transfer in nitric-oxide synthase. J. Biol. Chem..

[21] Venema R.C., Ju H., Zou R., Ryan J.W., Venema V.J. (1997). Subunit interactions of endothelial nitric-oxide synthase. Comparisons to the neuronal and inducible nitric-oxide synthase isoforms. J. Biol. Chem..

[22] Fang J., Ji H., Lawton G.R., Xue F., Roman L.J., Silverman R.B. (2009). L337H mutant of rat neuronal nitric oxide synthase resembles human neuronal nitric oxide synthase toward inhibitors. J. Med. Chem..

[23] Bogdan C. (2001). Nitric oxide and the immune response. Nat. Immunol..

[24] Zhou X., Hollern D., Liao J., Andrechek E., Wang H. (2013). NMDA receptor-mediated excitotoxicity depends on the coactivation of synaptic and extrasynaptic receptors. Cell Death Dis..

[25] Bradley S.A., Steinert J.R. (2016). Nitric Oxide-Mediated Posttranslational Modifications: Impacts at the Synapse. Oxid. Med. Cell. Longev..

[26] Herrero M.T., Yuste J.E., Cuenca-Bermejo L., Almela P., Arenas-Betancur L., De Pablos V., Gonzalez-Cuello A., Del Bel E., Navarro-Zaragoza J., Fernández-Villalba E. (2023). 7-Nitroindazole reduces L-DOPA-induced dyskinesias in non-human Parkinsonian primate. Open Biol..

[27] Yang T., Guo R., Ofengeim D., Hwang J., Zukin R.S., Chen J., Zhang F., Grotta J.C., Albers G.W., Broderick J.P., Day A.L., Kasner S.E., Lo E.H., Sacco R.L., Wong L.K.S. (2022). 5–Molecular and Cellular Mechanisms of Ischemia-Induced Neuronal Death; In Stroke.

[28] O‘Toole E., Doucet M.V., Sherwin E., Harkin A., Thomas F. (2016). Chapter 3—Novel Targets in the Glutamate and Nitric Oxide Neurotransmitter Systems for the Treatment of Depression. Systems Neuroscience in Depression.

[29] Wolff D.J., Gribin B.J. (1994). The inhibition of the constitutive and inducible nitric oxide synthase isoforms by indazole agents. Arch. Biochem. Biophys..

[30] Reiner A., Zagvazdin Y. (1998). On the selectivity of 7-nitroindazole as an inhibitor of neuronal nitric oxide synthase. Trends Pharmacol. Sci..

[31] Xue F., Li H., Delker S.L., Fang J., Martásek P., Roman L.J., Poulos T.L., Silverman R.B. (2010). Potent, highly selective, and orally bioavailable gem-difluorinated monocationic inhibitors of neuronal nitric oxide synthase. J. Am. Chem. Soc..

[32] Kang S., Tang W., Li H., Chreifi G., Martásek P., Roman L.J., Poulos T.L., Silverman R.B. (2014). Nitric oxide synthase inhibitors that interact with both heme propionate and tetrahydrobiopterin show high isoform selectivity. J. Med. Chem..

[33] Vasu D., Do H.T., Li H., Hardy C.D., Awasthi A., Poulos T.L., Silverman R.B. (2023). Potent, Selective, and Membrane Permeable 2-Amino-4-Substituted Pyridine-Based Neuronal Nitric Oxide Synthase Inhibitors. J. Med. Chem..

[34] Mukherjee P., Cinelli M.A., Kang S., Silverman R.B. (2014). Development of nitric oxide synthase inhibitors for neurodegeneration and neuropathic pain. Chem. Soc. Rev..

[35] Li H., Hardy C.D., Reidl C.T., Jing Q., Xue F., Cinelli M., Silverman R.B., Poulos T.L. (2024). Crystallographic and Computational Insights into Isoform-Selective Dynamics in Nitric Oxide Synthase. Biochemistry.

[36] Bach A., Clausen B.H., Kristensen L.K., Andersen M.G., Ellman D.G., Hansen P.B.L., Hasseldam H., Heitz M., Ozcelik D., Tuck E.J. (2019). Selectivity, efficacy and toxicity studies of UCCB01-144, a dimeric neuroprotective PSD-95 inhibitor. Neuropharmacology.

[37] Bach A., Clausen B.H., Møller M., Vestergaard B., Chi C.N., Round A., Sørensen P.L., Nissen K.B., Kastrup J.S., Gajhede M. (2012). A high-affinity, dimeric inhibitor of PSD-95 bivalently interacts with PDZ1-2 and protects against ischemic brain damage. Proc. Natl. Acad. Sci. USA.

[38] Zhou L., Li F., Xu H.B., Luo C.X., Wu H.Y., Zhu M.M., Lu W., Ji X., Zhou Q.G., Zhu D.Y. (2010). Treatment of cerebral ischemia by disrupting ischemia-induced interaction of nNOS with PSD-95. Nat. Med..

[39] Wu H., Huang Z., Wang X., Chen M., Chen W., Hua Y., Ren J., Shen L., Song Y., Zhou Y. (2023). Preclinical evaluation of ZL006-05, a new antistroke drug with fast-onset antidepressant and anxiolytic effects. Stroke Vasc. Neurol..

[40] Hu W., Guan L.S., Dang X.B., Ren P.Y., Zhang Y.L. (2014). Small-molecule inhibitors at the PSD-95/nNOS interface attenuate MPP+-induced neuronal injury through Sirt3 mediated inhibition of mitochondrial dysfunction. J. Neurochem. Int..

[41] Mo S.F., Liao G.Y., Yang J., Wang M.Y., Hu Y., Lian G.N., Kong L.D., Zhao Y. (2016). Protection of neuronal cells from excitotoxicity by disrupting nNOS-PSD95 interaction with a small molecule SCR-4026. Brain Res..

[42] Zhou X.F. (2021). ESCAPE-NA1 Trial Brings Hope of Neuroprotective Drugs for Acute Ischemic Stroke: Highlights of the Phase 3 Clinical Trial on Nerinetide. Neurosci. Bull..

[43] Chen W., Jiang B., Zhao Y., Yu W., Zhang M., Liang Z., Liu X., Ye B., Chen D., Yang L. (2023). Discovery of benzyloxy benzamide derivatives as potent neuroprotective agents against ischemic stroke. Eur. J. Med. Chem..

[44] Huang J.B., Chen Z.R., Yang S.L., Hong F.F. (2023). Nitric Oxide Synthases in Rheumatoid Arthritis. Molecules.

[45] Kolios G., Valatas V., Ward S.G. (2004). Nitric oxide in inflammatory bowel disease: A universal messenger in an unsolved puzzle. Immunology.

[46] Prado C.M., Martins M.A., Tibério I.F. (2011). Nitric oxide in asthma physiopathology. ISRN Allergy.

[47] Steinert J.R., Chernova T., Forsythe I.D. (2010). Nitric Oxide Signaling in Brain Function, Dysfunction, and Dementia. Neuroscientist.

[48] Maccallini C., Gallorini M., Cataldi A., Amoroso R. (2020). Targeting iNOS As a Valuable Strategy for the Therapy of Glioma. ChemMedChem.

[49] Kielbik M., Szulc-Kielbik I., Klink M. (2019). The Potential Role of iNOS in Ovarian Cancer Progression and Chemoresistance. Int. J. Mol. Sci..

[50] Kostourou V., Cartwright J.E., Johnstone A.P., Boult J.K., Cullis E.R., Whitley G., Robinson S.P. (2011). The role of tumour-derived iNOS in tumour progression and angiogenesis. Br. J. Cancer..

[51] Maccallini C., Patruno A., Besker N., Alì J.I., Ammazzalorso A., De Filippis B., Franceschelli S., Giampietro L., Pesce M., Reale M. (2009). Synthesis, biological evaluation, and docking studies of N-substituted acetamidines as selective inhibitors of inducible nitric oxide synthase. J. Med. Chem..

[52] Alderton W.K., Angell A.D., Craig C., Dawson J., Garvey E., Moncada S., Monkhouse J., Rees D., Russell L.J., Russell R.J. (2005). GW274150 and GW273629 are potent and highly selective inhibitors of inducible nitric oxide synthase in vitro and in vivo. Br. J. Pharmacol..

[53] Grant S.K., Green B.G., Stiffey-Wilusz J., Durette P.L., Shah S.K., Kozarich J.W. (1998). Structural requirements for human inducible nitric oxide synthase substrates and substrate analogue inhibitors. Biochemistry.

[54] Pérez-Asensio F.J., Hurtado O., Burguete M.C., Moro M.A., Salom J.B., Lizasoain I., Torregrosa G., Leza J.C., Alborch E., Castillo J. (2005). Inhibition of iNOS activity by 1400W decreases glutamate release and ameliorates stroke outcome after experimental ischemia. Neurobiol. Dis..

[55] Tang Q., Svensson C.I., Fitzsimmons B., Webb M., Yaksh T.L., Hua X.Y. (2007). Inhibition of spinal constitutive NOS-2 by 1400W attenuates tissue injury and inflammation-induced hyperalgesia and spinal p38 activation. Eur. J. Neurosci..

[56] Merenzon M.A., Arias E.H., Bhatia S., Shah A.H., Higgins D.M.O., Villaverde M., Belgorosky D., Eijan A.M. (2023). Nitric oxide synthase inhibitors as potential therapeutic agents for gliomas: A systematic review. Nitric Oxide.

[57] Strub A., Ulrich W.R., Hesslinger C., Eltze M., Fuchss T., Strassner J., Strand S., Lehner M.D., Boer R. (2006). The novel imidazopyridine 2-[2-(4-methoxy-pyridin-2-yl)-ethyl]-3H-imidazo[4,5-b]pyridine (BYK191023) is a highly selective inhibitor of the inducible nitric-oxide synthase. Mol. Pharmacol..

[58] Zahmatkesh M., Kadkhodaee M., Arab H.A., Shams S. (2006). Effects of co-administration of an iNOS inhibitor with a broad-spectrum reactive species scavenger in rat renal ischemia/reperfusion injury. Nephron Exp. Nephrol..

[59] Su F., Huang H., Akieda K., Occhipinti G., Donadello K., Piagnerelli M., De Backer D., Vincent J.L. (2010). Effects of a selective iNOS inhibitor versus norepinephrine in the treatment of septic shock. Shock.

[60] Broom L., Marinova-Mutafchieva L., Sadeghian M., Davis J.B., Medhurst A.D., Dexter D.T. (2011). Neuroprotection by the selective iNOS inhibitor GW274150 in a model of Parkinson disease. Free Radic. Biol. Med..

[61] Seymour M., Pétavy F., Chiesa F., Perry H., Lukey P.T., Binks M., Donatien P.D., Freidin A.J., Eckersley R.J., McClinton C. (2012). Ultrasonographic measures of synovitis in an early phase clinical trial: A double-blind, randomised, placebo and comparator controlled phase IIa trial of GW274150 (a selective inducible nitric oxide synthase inhibitor) in rheumatoid arthritis. Clin. Exp. Rheumatol..

[62] Barbanti P., Egeo G., Aurilia C., Fofi L., Della-Morte D. (2014). Drugs targeting nitric oxide synthase for migraine treatment. Expert. Opin. Investig. Drugs.

[63] Høivik H.O., Laurijssens B.E., Harnisch L.O., Twomey C.K., Dixon R.M., Kirkham A.J., Williams P.M., Wentz A.L., Lunnon M.W. (2010). Lack of efficacy of the selective iNOS inhibitor GW274150 in prophylaxis of migraine headache. Cephalalgia.

[64] Dao V.T., Elbatrik M., Fuchß T., Grädler U., Schmidt H., Shah A.M., Knowles R., Schmidt H.H.H.W., Ghezzi P., Cuadrado A. (2021). Nitric Oxide Synthase Inhibitors into the Clinic at Last. Handbook of Experimental Pharmacology No. 1.

[65] Carrión M.D., Rubio-Ruiz B., Franco-Montalban F., Amoia P., Zuccarini M.C., De Simone C., Camacho M.E., Amoroso R., Maccallini C. (2023). New amidine-benzenesulfonamides as iNOS inhibitors for the therapy of the triple negative breast cancer. Eur. J. Med. Chem..

[66] Mohamed M.F.A., Marzouk A.A., Nafady A., El-Gamal D.A., Allam R.M., Abuo-Rahma G.E.A., El Subbagh H.I., Moustafa A.H. (2020). Design, synthesis and molecular modeling of novel aryl carboximidamides and 3-aryl-1,2,4-oxadiazoles derived from indomethacin as potent anti-inflammatory iNOS/PGE2 inhibitors. Bioorg. Chem..

[67] Ibrahim T.S., Moustafa A.H., Almalki A.J., Allam R.M., Althagafi A., Md S., Mohamed M.F.A. (2021). Novel chalcone/aryl carboximidamide hybrids as potent anti-inflammatory via inhibition of prostaglandin E2 and inducible NO synthase activities: Design, synthesis, molecular docking studies and ADMET prediction. J. Enzyme Inhib. Med. Chem..

[68] Guillon C., Vetrano A.M., Saxena J., Hunter A., Verderone G., Finetti T.M., Wisnoski J., DeMatteo P.W., Rapp R.D., Heindel N.D. (2020). Derivatives of 1,2,4-triazole imines acting as dual iNOS and tumor cell growth inhibitors. Bioorg. Chem..

[69] Ahmed A.H.H., Mohamed M.F.A., Allam R.M., Nafady A., Mohamed S.K., Gouda A.E., Beshr E.A.M. (2022). Design, synthesis, and molecular docking of novel pyrazole-chalcone analogs of lonazolac as 5-LOX, iNOS and tubulin polymerization inhibitors with potential anticancer and anti-inflammatory activities. Bioorg. Chem..

[70] Yoon S.H., Cho D.Y., Han J.H., Choi D.K., Kim E., Park J.Y. (2023). Synthesis of 1-(5-(4-chlorophenyl)-1-(2,4-dichlorophenyl)-4-methyl-1H-pyrazol-3-yl)-2-morpholinoethane-1,2-dione analogues and their inhibitory activities with reduced cytotoxicity in lipopolysaccharide-induced BV2 cells. Bioorg. Med. Chem. Lett..

[71] Elkamhawy A., Hassan A.H.E., Paik S., Sup Lee Y., Lee H.H., Shin J.S., Lee K.T., Roh E.J. (2019). EGFR inhibitors from cancer to inflammation: Discovery of 4-fluoro-N-(4-(3-(trifluoromethyl)phenoxy)pyrimidin-5-yl)benzamide as a novel anti-inflammatory EGFR inhibitor. Bioorg. Chem..

[72] Shi J.B., Chen L.Z., Wang B.S., Huang X., Jiao M.M., Liu M.M., Tang W.J., Liu X.H. (2019). Novel Pyrazolo[4,3- d]pyrimidine as Potent and Orally Active Inducible Nitric Oxide Synthase (iNOS) Dimerization Inhibitor with Efficacy in Rheumatoid Arthritis Mouse Model. J. Med. Chem..

[73] Chen L.Z., Shu H.Y., Wu J., Yu Y.L., Ma D., Huang X., Liu M.M., Liu X.H., Shi J.B. (2021). Discovery and development of novel pyrimidine and pyrazolo/thieno-fused pyrimidine derivatives as potent and orally active inducible nitric oxide synthase dimerization inhibitor with efficacy for arthritis. Eur. J. Med. Chem..

[74] Zhu L.Q., Fan X.H., Li J.F., Chen J.H., Liang Y., Hu X.L., Ma S.M., Hao X.Y., Shi T., Wang Z. (2021). Discovery of a novel inhibitor of nitric oxide production with potential therapeutic effect on acute inflammation. Bioorg. Med. Chem. Lett..

[75] Karbach S., Wenzel P., Waisman A., Munzel T., Daiber A. (2014). eNOS uncoupling in cardiovascular diseases--the role of oxidative stress and inflammation. Curr. Pharm. Des..

[76] Kietadisorn R., Juni R.P., Moens A.L. (2012). Tackling endothelial dysfunction by modulating NOS uncoupling: New insights into its pathogenesis and therapeutic possibilities. Am. J. Physiol. Endocrinol. Metab..

[77] Shaito A., Aramouni K., Assaf R., Parenti A., Orekhov A., Yazbi A.E., Pintus G., Eid A.H. (2022). Oxidative Stress-Induced Endothelial Dysfunction in Cardiovascular Diseases. Front. Biosci..

[78] Fraccarollo D., Widder J.D., Galuppo P., Thum T., Tsikas D., Hoffmann M., Ruetten H., Ertl G., Bauersachs J. (2008). Improvement in left ventricular remodeling by the endothelial nitric oxide synthase enhancer AVE9488 after experimental myocardial infarction. Circulation.

[79] Yang Q., Xue H.M., Wong W.T., Tian X.Y., Huang Y., Tsui S.K., Ng P.K., Wohlfart P., Li H., Xia N. (2011). AVE3085, an enhancer of endothelial nitric oxide synthase, restores endothelial function and reduces blood pressure in spontaneously hypertensive rats. Br. J. Pharmacol..

[80] Cheang W.S., Wong W.T., Tian X.Y., Yang Q., Lee H.K., He G.W., Yao X., Huang Y. (2011). Endothelial nitric oxide synthase enhancer reduces oxidative stress and restores endothelial function in db/db mice. Cardiovasc. Res..

[81] Heitzer T., Brockhoff C., Mayer B., Warnholtz A., Mollnau H., Henne S., Meinertz T., Münzel T. (2000). Tetrahydrobiopterin improves endothelium-dependent vasodilation in chronic smokers: Evidence for a dysfunctional nitric oxide synthase. Circ. Res..

[82] Daiber A., Steven S., Weber A., Shuvaev V.V., Muzykantov V.R., Laher I., Li H., Lamas S., Münzel T. (2017). Targeting vascular (endothelial) dysfunction. Br. J. Pharmacol..

[83] Serreli G., Deiana M. (2023). Role of Dietary Polyphenols in the Activity and Expression of Nitric Oxide Synthases: A Review. Antioxidants.

[84] Song X., Tan L., Wang M., Ren C., Guo C., Yang B., Ren Y., Cao Z., Li Y., Pei J. (2021). Myricetin: A review of the most recent research. Biomed. Pharmacother..

[85] Hu W.H., Wang H.Y., Xia Y.T., Dai D.K., Xiong Q.P., Dong T.T., Duan R., Chan G.K., Qin Q.W., Tsim K.W. (2020). Kaempferol, a Major Flavonoid in Ginkgo Folium, Potentiates Angiogenic Functions in Cultured Endothelial Cells by Binding to Vascular Endothelial Growth Factor. Front. Pharmacol..

[86] Li P.G., Sun L., Han X., Ling S., Gan W.T., Xu J.W. (2012). Quercetin induces rapid eNOS phosphorylation and vasodilation by an Akt-independent and PKA-dependent mechanism. Pharmacology.

[87] Dagher O., Mury P., Thorin-Trescases N., Noly P.E., Thorin E., Carrier M. (2021). Therapeutic Potential of Quercetin to Alleviate Endothelial Dysfunction in Age-Related Cardiovascular Diseases. Front. Cardiovasc. Med..

[88] Zhang S., Hu X., Guo S., Shi L., He Q., Zhang P., Yu S., Zhao R. (2019). Myricetin ameliorated ischemia/reperfusion-induced brain endothelial permeability by improvement of eNOS uncoupling and activation eNOS/NO. J. Pharmacol. Sci..

[89] Seong E.H., Gong D.S., Shiwakoti S., Adhikari D., Kim H.J., Oak M.H. (2022). Taxifolin as a Major Bioactive Compound in the Vasorelaxant Effect of Different Pigmented Rice Bran Extracts. Front. Pharmacol..

[90] Xia N., Daiber A., Habermeier A., Closs E.I., Thum T., Spanier G., Lu Q., Oelze M., Torzewski M., Lackner K.J. (2010). Resveratrol reverses endothelial nitric-oxide synthase uncoupling in apolipoprotein E knockout mice. J. Pharmacol. Exp. Ther..

[91] Duarte J., Francisco V., Perez-Vizcaino F. (2014). Modulation of nitric oxide by flavonoids. Food Funct..

[92] Pereira S.C., Cotta Filho C.K., Lacchini R. (2021). The need for further studies examining the role of endothelial nitric oxide synthase polymorphisms in drug response. Pharmacogenomics.

